# Efficacy and safety of Brivaracetam as an adjunctive therapy in patients with focal-onset seizures: a phase III, multi-center, randomized, double-blind, placebo-controlled trial

**DOI:** 10.1186/s42494-026-00253-7

**Published:** 2026-05-07

**Authors:** Peimin Yu, Ting Zhao, Weihong Lin, Xiong Han, Feng Li, Zhen Hong

**Affiliations:** 1 Department of Neurology, Huashan Hospital, Fudan University, No.12, Middle Wulumuqi Road, Jing’an District, Shanghai, 200040 China; 2 Department of Neurology, Henan Provincial People’s Hospital, Zhengzhou University People’s Hospital, No.7, Wei Wu Road, Jinshui District, Zhengzhou, Henan 450003 China; 3 Department of Neurology, First Hospital of Jilin University, No.1 Xinmin Street, Chaoyang District, Changchun, Jilin 130000 China; 4 Department of Neurology, The First Affiliated Hospital of Chongqing Medical University, No.1, Youyi Road, Yuanjiagang Road, Yuzhong District, Chongqing, 400016 China

**Keywords:** Brivaracetam, Focal-onset epilepsy, Seizure frequency, Responder rate, Randomized control trial

## Abstract

**Background:**

Preclinical experiments have revealed that Brivaracetam (BRV) exhibited a significant anti-seizure function in animal models and was more effective and safer than other synaptic vesicle protein 2A (SV2A) ligands, including levetiracetam (LEV), this study aims to evaluate the efficacy and safety of generic BRV tablets in Chinese patients with focal-onset seizure.

**Methods:**

This phase III, randomized, double-blind, placebo-controlled clinical trial conducted in 21 medical centers evaluated BRV (200 mg/day) as an adjunctive therapy in Chinese adult patients with focal-onset seizures, with or without secondary generalization, despite treatment with one or two permitted concomitant anti-seizure medications (ASMs). After an 8-week screening baseline, patients were 1:1 randomized to BRV 200 mg/day or placebo for a 12-week treatment period. The primary efficacy endpoint was the percent reduction in seizure frequency per 28 days from baseline. Safety evaluations included the adverse events (AEs), side effects, and the regular monitoring of clinical symptoms, vital signs, physical examinations, laboratory tests, electrocardiograms, and mood.

**Results:**

Of 179 randomized patients, 178 were included in the final analysis (90 in BRV; 88 in placebo), while one patient in placebo group was excluded for not receiving the study drug. The percent reduction in seizure frequency per 28 days from baseline was 40.67% in the BRV group compared to the placebo group. The 12-week responder rate was significantly higher with BRV than placebo [48.89% vs. 23.86%, Odds Ratio (OR) = 3.12, 95% Confidence Interval (CI): 1.63–5.99, *P* = 0.0006]. There's no statistical significance of reported AEs in the BRV and placebo groups. Seizure freedom was achieved by 10 (11.11%) and 2 (2.27%) patients in the BRV and placebo groups, respectively (*P* = 0.0325). Discontinuations due to AEs (6.67% vs. 0, *P* = 0.0287) and special concern AEs (32.22% vs. 9.09%, *P* = 0.0002) were higher with BRV than placebo. Dizziness, somnolence, and nausea were reported more frequently with BRV than placebo.

**Conclusions:**

Generic BRV demonstrated a better efficacy and comparable safety in treating focal-onset seizure in Chinese patients.

## Background

Epilepsy is a chronic brain disorder characterized by recurrent and unprovoked seizures, and is the second commonest disorder in the central nervous system. Globally, there are about 50 million people with epilepsy. Epilepsy causes neurological, cognitive, psychological, and social consequences and accounts for a significant proportion of the world’s burden of disease [1]. There are various types of seizures, each differing in intensity, but one of the most common types in adults is focal-onset seizures, which may affect awareness, movement, and sensation [2]. Up to date, anti-seizure medications (ASMs) have remained suboptimal for one-third of patients who either still have refractory seizures or suffer from side effects [3]. This emphasizes the priority of developing new ASMs that are more effective and better tolerated.

Brivaracetam (BRV) is a newly developed ASM poised to be more effective in treatment than the other options available in the market. It is a high-affinity ligand for the synaptic vesicle protein 2 A (SV2A), involved in neurotransmitter release. BRV interacts with SV2A protein which is involved in the cycling of synaptic vesicles in neurons and achieve the anti-seizure action [4]. Previous preclinical experiments have revealed that BRV exhibited a significant anti-seizure function in animal models and was more effective and safer than other SV2A ligands, including levetiracetam (LEV) [5]. Previous clinical trials and real-world observations were conducted mostly in high income countries and yielded more information regarding the efficacy of BRV in treating focal-onset seizures [6–8]. However, evidence in large-sampled Chinese patients is still lacking.

A more pressing challenge is the profound disparity in ASM access between high-income countries and low- and middle-income countries (LMICs), where over 80% of people with epilepsy reside. Proprietary ASMs remain prohibitively expensive for most patients in these settings, with costs representing a major barrier to treatment initiation and continuation. Generic alternatives offer the only viable pathway to sustainable, equitable epilepsy care in LMICs, yet rigorous efficacy and safety data in local populations are essential for regulatory approval and clinical confidence. This study addresses both needs simultaneously by evaluating a generic BRV formulation specifically within the Chinese healthcare context.

This clinical trial aimed to provide a comprehensive assessment of the efficacy and tolerability of BRV tablets in Chinese patients with focal-onset seizure. The hypothesis of this study was to verify that BRV was more effective than placebo in controlling focal-onset seizures. This hypothesis was based on the earlier findings of the pharmacodynamics and pharmacokinetics of the drug, cell cultures, animal studies, and early case reports.

## Methods

### Study design

This was a phase III, randomized, double-blind, placebo-controlled clinical trial (http://www.chinadrugtrials.org.cn, CTR20202124) conducted in 21 medical centers in China. This therapeutic confirmatory trial evaluated the generic drug BRV (200 mg/day) as a treatment in Chinese adult patients with focal-onset seizures with or without secondary generalization despite current treatment with one or two permitted concomitant ASMs.

The trial was conducted in accordance with the International Conference on Harmonization Good Clinical Practice requirements and the Declaration of Helsinki.The study protocol, amendments, and informed consent were approved by the local Ethics Committee in each center (Approval number: [2020] Lin-Shen No. 853). Written informed consents were obtained from patients or their legally authorized representatives.

### Study participants

Eligible patients were aged ≥ 16 and ≤ 80 years with a history of uncontrolled focal-onset seizures according to the International League Against Epilepsy’s (ILAE) Classification of Epileptic Seizures (1981) [9], with or without secondary generalization. The diagnosis was established by clinical history, an electroencephalogram (EEG) within the last 5 years and brain MRI/CT scan within 6 months prior to screening. Inclusion criteria also included that the patient had to have ≥ 8 focal seizures in the 8 weeks prior to treatment and at least ≥ 2 focal seizures every 4 weeks, with no seizure interval ≤ 21 days. Focal seizures had not been controlled by stable dose using of 1–2 ASMs for at least 1 month prior to the screening (at least 3 months if using phenobarbital, phenytoin, or promidone). Stable using of permitted concomitant ASMs and vagus nerve stimulation (VNS) (as a concomitant ASM) was required for at least 1 month prior to the screening (at least 3 months for using phenobarbital, phenytoin, or primidone), and was required to be maintained throughout the trial. Benzodiazepines (for any indication) that used exceeding once a week was considered as a concomitant ASM. Patients were excluded if they met any of the following criteria. 1) only having non-motor simple focal seizures; 2) had status epilepticus in the past 1 year or at the baseline; 3) seizure frequency could not be counted due to the extremely high frequency; 4) diagnosed with psychogenic non-epileptic seizure or non-epileptic seizure; 5) had treated with BRV; 6) being treated with LEV or had been treated with LEV in 90 days prior to the screening; 7) being treated or had been treated with vigabatrin in 6 months prior to the screening; 8) had been treated with Chinese Traditional Medicine or ASMs containing any components of Chinese Traditional Medicine; 9) being treated with any drugs that may have an impact on the central nervous system (CNS) or the cytochrome P450 (potential inducers), which may influence the metabolism of Brivaracetam, unless they had been stable for at least 1 month prior to the screening and were expected to remain stable during the trial; 10) with a history of drug abuse (soft drugs such as marijuana in 90 days prior to the screening or hard drugs such as cocaine in 365 days prior to the screening); 11) allergic to pyrrolidine drugs such as BRV or LEV, or with a history of multiple (severe) allergies; 12) with a history of attempted suicide or suicidal ideation in 6 months prior to the screening; 13) had participated in another drug or medical device trial in the previous 3 months, or plan to participate in other clinical trials during the study period; 14) investigators believe that they had any severe medical or mental disorders that may not be appropriate to participate in this trial.

### Trial procedures

**Fig. 1:**
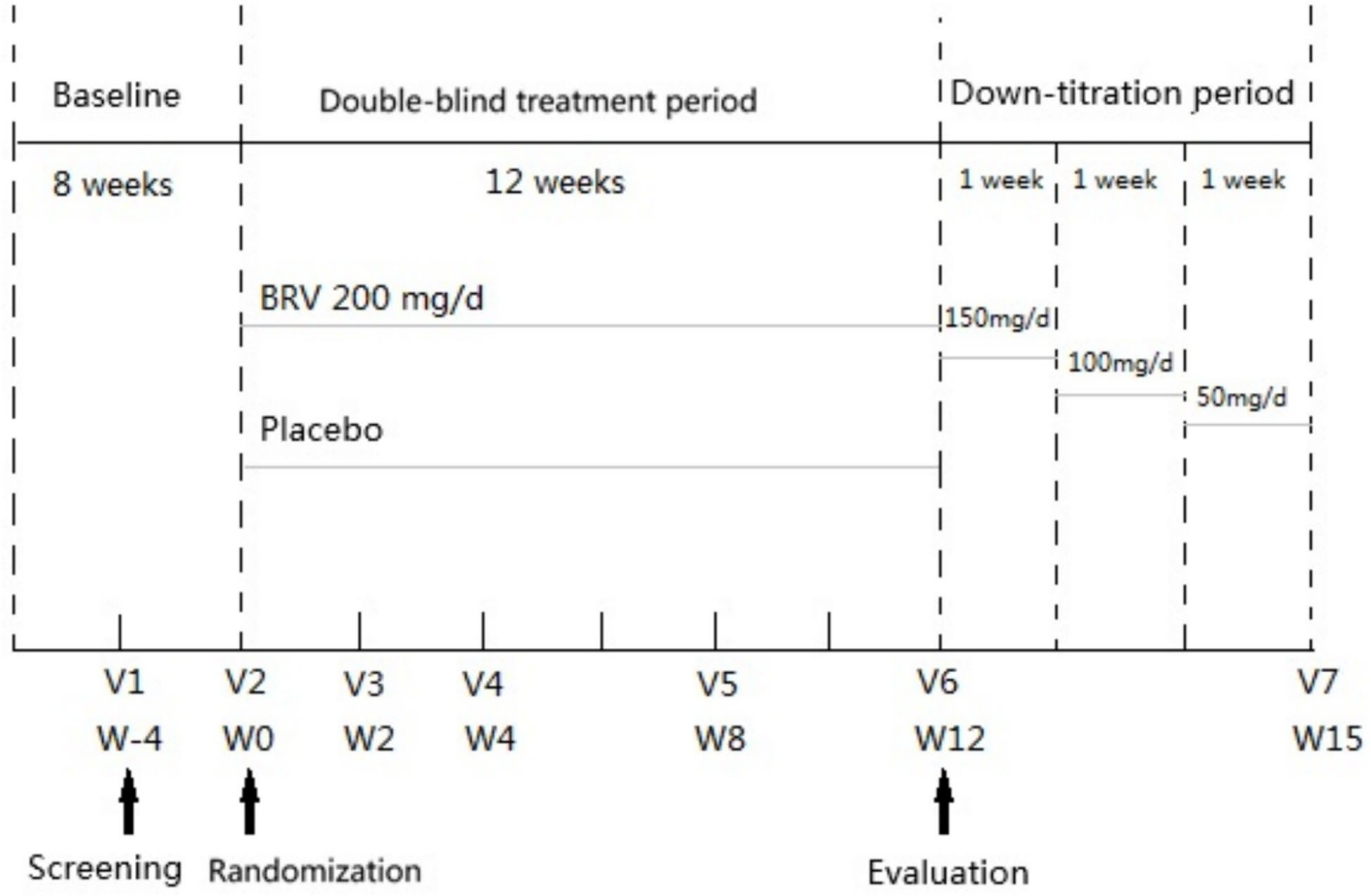
Procedure of clinical trial

This trial started with an 8-week baseline period, including a retrospective baseline of 4 weeks before the screening and a prospective baseline of 4 weeks after the screening. Participants were required to have seizure records in the retrospective period of 4 weeks. If not available, seizure records in a prospective baseline period of 8 weeks were required. After that, patients were 1:1 randomized to two treatment arms (BRV vs. placebo). The number of ASMs (≤ 2 or > 2) that had been used before but were no longer used after entering the trial was considered as a stratification factor in the randomization to ensure the balance between two treatment groups at baseline. In the 12-week double-blind treatment period, patients in the BRV group received BRV tablets with a dosage of 100 mg bisindie (bid) in the morning and evening, equal to 200 mg/day, while the placebo group received placebo with the dosage and treatment schedule matched with BRV. In the following a 4-week down-titration period, the dosages of either BRV or placebo were decreased to 150 mg/day (75 mg bid) at week 13, 100 mg/day (50 mg bid) at week 14, and 50 mg/day (25 mg bid) at week 15, respectively. Follow-up interviews were administered at week 2, week 4, week 8, and week 12 for efficacy and safety evaluations. Follow-up interview at week 15 were administered for safety evaluation. The design and schedule of this trial is shown in Fig. 1.

### Endpoints and outcome measures

Outcomes of efficacy were assessed over the 12-week treatment period based on the data collected at week 2, 4, 8, and 12. The primary endpoint of efficacy was the percent reduction of focal seizure frequency per 28 days from baseline. Secondary efficacy endpoints were: 1) the responder rate, defined as the percentage of patients who achieved 50% percent reduction of focal seizure frequency per 28-day from baseline; 2) the percent reduction of focal seizure frequency per 28-day from baseline; 3) the percentage of patients who achieved different level of percent reduction (−25% to < 25%, 25% to < 50%, 50% to < 75%, 75% to < 100%, and 100%) of focal seizure frequency per 28-day from baseline; 4) the time from initial treatment to the 1 st, 5th, and 10th onset of focal seizures; and 5) the percentage of patients who achieved seizure freedom during the complete treatment period.

Safety evaluations were based on the regular monitoring of clinical symptoms, vital signs, physical examinations, laboratory tests (blood routine, blood biochemistry, urine routine, pregnancy test), 12-ECG, Colombia-Suicide Severity Rating Scale (C-SSSRS) at each follow-up visit until week 15. Adverse events (AEs)/side effects (SEs) were determined based on the abnormal changes with clinical significance, severity, occurrence time, end time, treatment, outcomes, and the correlation with the study drugs. Definitions of special concern AEs/SEs were based on the guiding principles for clinical research and trial techniques of epilepsy treatment drugs established by the China Center for Drug Evaluation [10].

### Statistical analysis

The sample size was calculated based on the primary efficacy endpoint, which was the percent reduction of focal seizure frequency per 28-day from baseline of BRV compared to placebo. The parameters employed in this calculation were the difference of 25% of change in seizure frequency between the BRV group and the placebo group [5]. Since the frequency of epileptic seizures did not fit a normal distribution, the effect difference between the BRV group and the placebo group was −0.286 (SD 0.62) after the logarithmic transformation, which was equivalent to a difference of nearly 25% of change in seizure frequency (formula: [1-EXP (−0.286)] × 100%). Therefore 74 patients in each treatment group (148 in total) were required to detect such difference at the 0.05 significance level (two-tailed) with an 80% statistical power. Considering a 20% lost-to-follow up rate, 176 patients were required for this trial.

Based on the intention-to-treat (ITT) principle, the full analysis set (FAS) consisted of all randomized patients who received ≥ 1 dose of study drug and had ≥ 1 post baseline seizure diary record during the treatment period. The safety set (SS) consisted of all randomized patients who received ≥ 1 dose of study drug across the period from baseline to the end of the down-titration period. Continuous variables were presented as mean (SD) and categorical variables were presented as the number of cases (*n*) and the frequency (%). To compare the differences in variables between two treatment groups, the independent sample *t*-test or nonparametric test was used for continuous data, while the chi-square or Fisher’s exact test was adopted for categorical variables wherever appropriate. The comparison of the percent reduction in focal seizure frequency change from baseline between the two treatment groups was conducted by ANCOVA. In this model, the ''treatment group'' was assumed to have a fixed effect while the ''baseline seizure frequency'' was assumed to have a random effect. The least squares means were derived from the ANCOVA model and a two-side test was conducted to test the difference between the groups with a significance level of 0.05. The safety outcomes including the rates of AEs/SEs were summarized descriptively and compared between the groups using chi-square test or Fisher's exact test.

All *P* values and 95% Confidence Intervals (CIs) were estimated in a two-tailed style. Differences were considered to be statistically significant at *P* < 0.05. Statistical analysis was performed using SAS software version 9 (SAS Institute, Cary, North Carolina, USA).

## Results

**Fig. 2:**
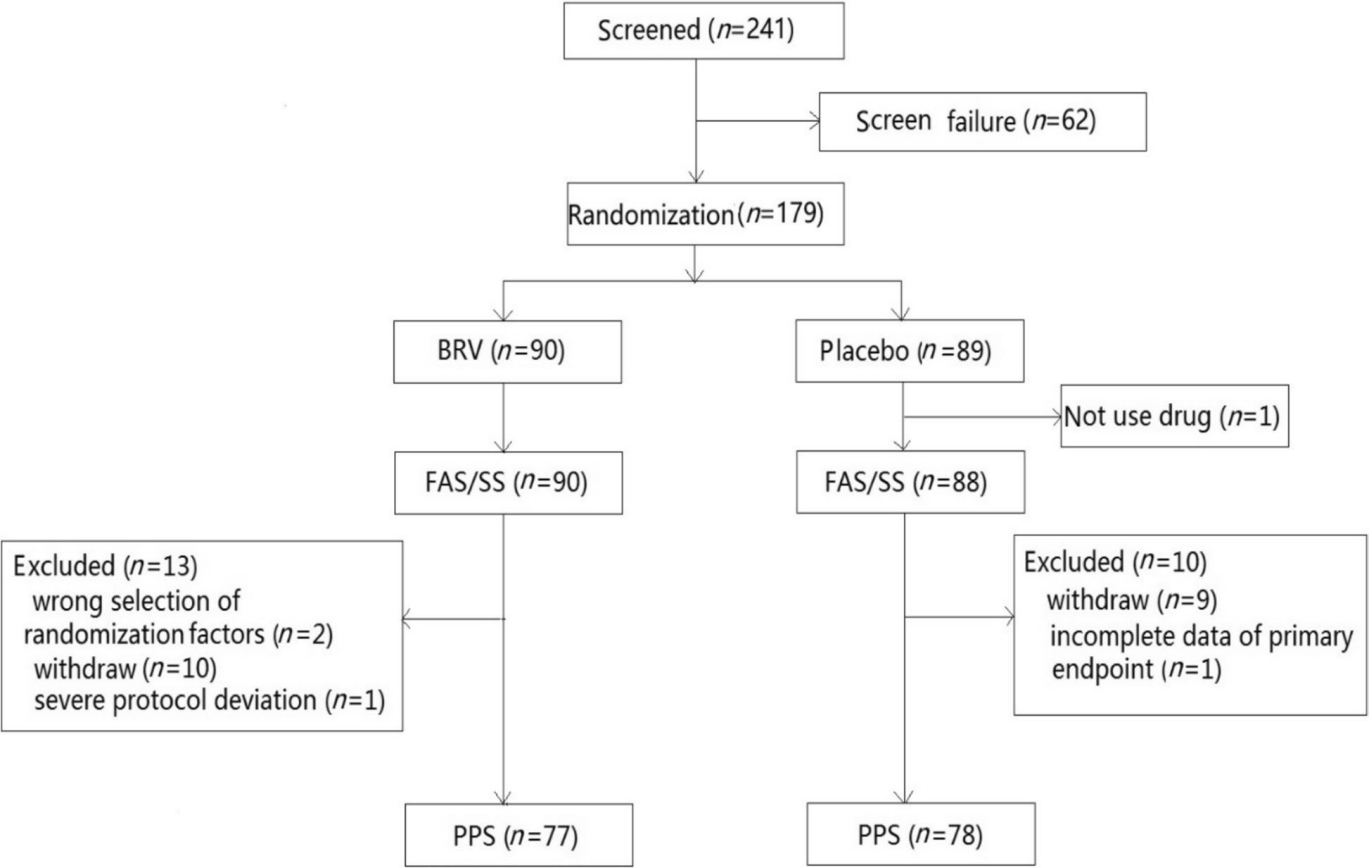
Flow-chart of the patients recruitment and datasets in the trial period. Note: BRV**,** Brivaracetam; FAS, Full Analysis Set; PPS, Per-Protocol Set; SS, Safety Set

As shown in Fig. 2, 241 patients were screened, among which 179 patients successfully passed the screening process and subsequently randomized into either the BRV or the placebo group. All patients in the BRV group commenced drug usage, whereas one patient in the placebo group did not use the drug. The FAS comprised 90 patients in the BRV group and 88 patients in the placebo group. Overall, 155 (87%) patients completed the trial with the discontinuation rates of 12.22% and 11.24% in the BRV and placebo groups. Four patients discontinued due to AEs and Severe Adverse Events (SAEs) in the BRV group, while one patient discontinued due to SAEs in the placebo group. Early withdrawal causes also included voluntary withdrawal of consent, pregnancy, treatment failures, protocol violations and other causes.

As shown in Table 1, there was no significant difference in demographics, epilepsy characteristics, medical history, and comorbidities between the two treatment groups (*P* > 0.05 for all comparisons). About 80% participants had clinically significant EEG abnormalities, and a substantial proportion of 50% had clinically significant brain CT/MRI findings. Over 60% of patients in both groups had used ≤ 2 ASMs prior to the trial, among whom about half had used one kind of ASM. The causes of epilepsy were predominantly unknown or unclear in about three-fourths of the patients. Other causes included intracranial infection, head trauma, brain surgery, cerebrovascular diseases, and other factors.

**Table 1:** Demographics and epilepsy characteristics of study participants at baseline

	Brivaracetam (*N* = 90)	Placebo (*N* = 88)	*P*-value
Age, mean ± SD, years	34.2 ± 12.4	33.4 ± 11.4	0.6176
Sex, male/female	55/35	42/46	0.0975
Nationality, Han Chinese/other	88/2	85/3	0.6804
BMI, mean ± SD, kg/m^2^	23.9 ± 4.3	23.8 ± 3.8	0.9142
Duration of Disease, mean ± SD, years	16.3 ± 10.8	17.0 ± 11.2	0.6418
Age of onset, mean ± SD, years	18.5 ± 13.6	16.9 ± 12.8	0.4156
No more than two ASMs previously used, *n* (%)	58 (64.4)	56 (63.6)	1.0000
Basic one ASM use, *n* (%)	30 (33.3)	28 (31.8)	0.8737
Levetiracetam use, *n* (%)	26 (28.9)	20 (22.7)	0.3940
Causes of epilepsy, unknown, *n* (%)	69 (76.7)	66 (75.0)	0.2833
Family history, *n* (%)	9 (10.0)	7 (8.0)	0.7944
Allergy history, *n* (%)	11 (12.2)	8 (9.1)	0.6288
Perinatal history, *n* (%)	12 (13.3)	8 (9.1)	0.4779
History of other vital diseases, *n* (%)	77 (85.6)	78 (88.6)	0.6563
Surgery history, *n* (%)	37 (41.1)	26 (29.6)	0.6189
EEG abnormalities, *n* (%)	77 (85.6)	70 (79.6)	0.4674
Brain CT/MRI abnormalities, *n* (%)	49 (54.4)	40 (45.5)	0.1485

**Fig. 3:**
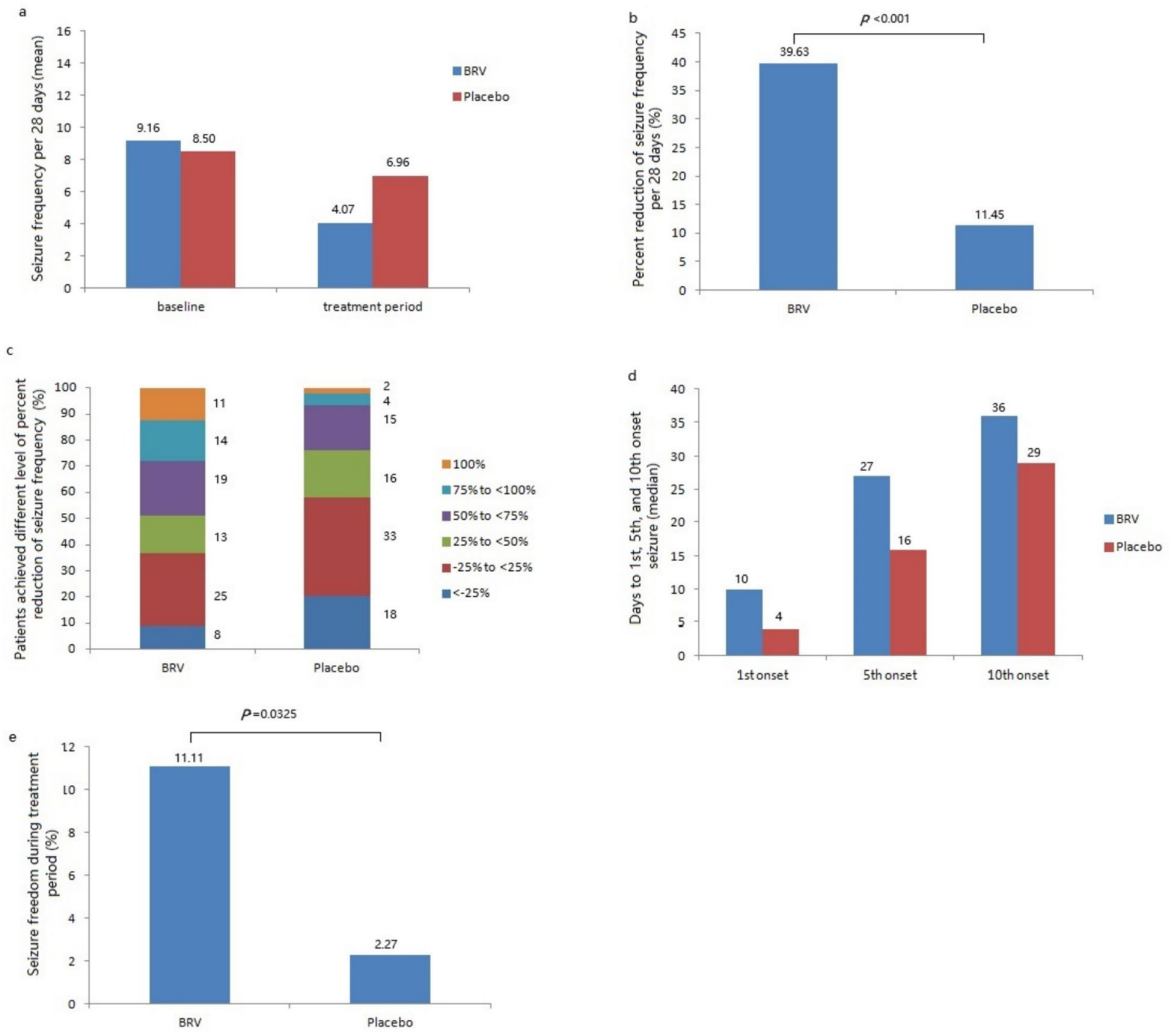
Efficacy analysis for the study participants (FAS): **a** 28-day focal-onset seizure frequency at baseline and during treatment period; **b** percent reduction in 28-day focal-onset seizure frequency during treatment period compared with baseline; **c** percentages of patients who achieved different level of percent reduction; **d** time to the 1 st, 5th, and 10th focal seizures (median days); **e** percentages of patients with seizure freedom from all seizure types during the treatment period

The median seizure frequency per 28 days in the BRV and placebo groups were 9.16 and 8.50 at baseline, and were 4.07 and 6.96 during the 12-week treatment period (Fig. 3a). The percent reduction in seizure frequency per 28 days from baseline was 40.67% in the BRV group compared to the placebo group. During the 12-week treatment period, the responder rate of BRV was significantly higher than placebo (48.89% vs. 23.86%, Odds Ratio [OR] = 3.12, 95% CI: 1.63–5.99, *P* = 0.0006). Almost 40% of the patients in the BRV group presented the reduction in focal seizure frequency per 28 days from baseline, which was significantly higher than that in the placebo group (39.63% vs. 11.45%, *P* < 0.0001) (Fig. 3b).Significant difference was found in the percentages of patients who achieved different level of percent reduction (−25% to < 25%, 25% to < 50%, 50% to < 75%, 75% to < 100%, and 100%) between two treatment groups (Fig. 3c). Days (median) to the 1 st, 5th, and 10th focal seizures were significantly longer in the Brivaracetam group compared to the placebo group (Fig. 3d). Seizure freedom was reported by 10 and 2 patients in the BRV and placebo groups respectively (11.11% vs. 2.27%, *P* = 0.0325) (Fig. 3e).

Table 2 summarized the incidence and symptoms of AEs/SEs during the whole period of trial. Sixty-three patients in the BRV group and 49 patients in the placebo group reported AEs but without statistical significance (70.00% vs. 55.68%, *P* = 0.0624). Discontinuations due to AEs (6.67% vs.0, *P* = 0.0287) and special concern AEs (32.22% vs. 9.09%, *P* = 0.0002) in the BRV were reported more than that in the placebo groups. SEs were reported by 47 patients in the BRV group, indicating a higher incidence compared to the placebo group (52.22% vs. 28.41, *P* = 0.0014). Among them, special concern SEs in the BRV were reported more than that in the placebo groups (30.00% vs. 7.95%, *P* = 0.0002). The most common (≥ 10%) AEs in patients receiving BRV were dizziness (23.33% vs. 7.95%, *P* = 0.0068) and somnolence (13.33% vs. 3.41%, *P* = 0.0281), which were significantly higher than that in patients receiving placebo. Nausea was reported in seven patients only in the BRV group (7.78% vs. 0, *P* = 0.0139). No significant difference was found in other safety measurement indexes between the two treatment groups, i.e. laboratory parameters, vital signs, body weight, ECG, physical examinations, or psychiatric status.

**Table 2:** Incidence of adverse events and side effects

	Brivaracetam *n* (%)	Placebo *n* (%)	*P-*value
*N*	90	88	
Adverse events	63 (70.00)	49 (55.68)	0.0624
Severe adverse events	2 (2.22)	2 (2.27)	1.0000
Discontinuation due to adverse events	6 (6.67)	0 (0.00)	0.0287
Special concern adverse events	29 (32.22)	8 (9.09)	0.0002
Adverse events ≥ level 3	3 (3.33)	2 (2.27)	1.0000
Adverse events leading to death	0 (0.00)	0 (0.00)	-
Adverse events ≥ 5% patients			
Dizziness	21 (23.33)	7 (7.95)	0.0068
Somnolence	12 (13.33)	3 (3.41)	0.0281
Urinary tract infection	9 (10.00)	11 (12.50)	0.6414
Nausea	7 (7.78)	0 (0.00)	0.0139
Drowsiness	6 (6.67)	3 (3.41)	0.4967
Fatigue	6 (6.67)	1 (1.14)	0.1178
Upper respiratory tract infection	4 (4.44)	8 (9.09)	0.2461
Hyperuricemia	3 (3.33)	5 (5.68)	0.4940
Side effects	47 (52.22)	25 (28.41)	0.0014
Severe side effects	2 (2.22)	0 (0.00)	0.4972
Discontinuation due to side effects	4 (4.44)	0 (0.00)	0.1209
Special concern side effects	27 (30.00)	7 (7.95)	0.0002
Side effects ≥ level 3	2 (2.22)	0 (0.00)	0.4972
Side effects leading to death	0 (0.00)	0 (0.00)	-
Side effects ≥ 5% patients			
Agitation	1 (1.11)	0 (0.00)	1.0000
Epileptic psychosis	1 (1.11)	0 (0.00)	1.0000
Cardiovascular somatic symptom disorder	0 (0.00)	1 (1.14)	0.4944
Toxicity of formulations	0 (0.00)	1 (1.14)	0.4944

## Discussion

This multi-center, randomized, double-blind, placebo-controlled clinical trial demonstrated the efficacy and safety of BRV (200 mg/day) in Chinese patients with focal-onset seizures. Compared with the original product, the BRV tablet as a generic drug in this study showed a better efficacy and similar safety on the treatment of focal-onset seizure, especially in Chinese patients. The development of generic BRV may reduce health care costs and improve drug accessibility in low-and middle-income countries.

In the current study, the percent reduction in seizure frequency per 28 days was about 40% in the BRV group compared to the placebo group during the treatment period. The responder rate (defined as at least 50% reduction of seizure frequency) was higher in the BRV group than that in the placebo group with the odds ratio of 3.12 (48.89% vs. 23.86%). These results are consistent with a multi-centre clinical trial reporting 33.4% as the percent reduction in seizure frequency per 28 days, and 49.3% as the 50% responder rate in Asian patients treated by BRV 200 mg/day [8]. A multi-center study with patients recruited from North America, Western Europe, Eastern Europe, Latin America, and Asia reported that, for BRV 200 mg/day, the percent reduction in 28-day seizure frequency was 23.2% over placebo, and the odds ratio of ≥ 50% responder rate was 2.19 comparing with placebo [5]. A pooled analysis of three clinical studies also indicated that the reduction over placebo in seizure frequency per 28 days was 24.0% for BRV 200 mg/day, and the ≥ 50% responder rate was 37.8% vs. 20.3% for placebo. Therefore, BRV had shown a favourable tolerability profile and a high responder rate in patients with focal-onset seizures [11].

The current trial also reported that BRV had acceptable tolerability and the AEs were manageable. Dizziness, somnolence, and nausea were more commonly reported in the BRV group than in the placebo group. Most AEs in the current trial were similar to those in previous BRV studies, affirming that BRV is safe and well tolerated [5, 12–18].

It was indicated that the anti-seizure effects of BRV might come from its strong binding to the SV2A. As the SV2A receptor agonist, BRV enhances the availability of neurotransmitters and allows the neuron to have optimal activity, which avoids seizures. BRV presents a higher efficacy than LEV although it is characterized by 15–30 fold lower binding affinity of SV2A than that of LEV [4, 5]. Pharmacokinetic evaluations also demonstrated that plasma concentrations of BRV were consistent across the treatment period, indicating consistent absorption and a dose-proportional increase in BRV plasma concentration [19].

There were some limitations in this trial which needs to be considered when interpreting the results. First, there may be some recall bias [20] when the patients were asked for recording the seizure frequency and seizure type data, especially at the screening phase. Second, the treatment and follow-up period was relatively short, thus the long-term efficacy and safety of BRV could not be observed and evaluated. Third, the study evaluated the safety measurements without detailed mood, cognitive function assessments, and quality of life evaluation [21, 22]. Fourth, only one fixed dose (200 mg/day) was tested in our study with titration or dose-ranging lacking. Fifth, LEV users were excluded from the study, while in real-world clinical practice in China and LMICs, LEV is one of the most widely prescribed ASMs. Sixth, the efficacy of BRV in the current study (e.g., 40.67% reduction over placebo) appears higher compared to some previous international trials. Potential reasons may include the different recruitment criteria, concomitant ASMs, or genetic factors of the study participants. Seventh, the ILAE Classification of Epileptic Seizures (1981) [9] was used instead of the current ILAE frameworks (recent 2025, or at least 2017). Lastly, the current trial only evaluated the mono-therapy of BRV vs. placebo, and the adjunctive effect of BRV with other ASMs should also be assessed. These factors mentioned above may limit the generalizability of the study findings.

## Conclusions

This study demonstrated the efficacy and tolerability of BRV (200 mg/day) as the treatment of focal-onset epilepsy in Chinese patients with epilepsy. The high percent reduction of seizure frequency, the high responder rate, and the safety profile made this generic BRV a valuable asset in the therapeutic tools for epilepsy management. More clinical trials and real-world studies with larger sample size and longer follow-up are needed to evaluate not only the anti-seizure efficacy of BRV, but also other consequences such as mood, cognitive function, and quality of life in patients with epilepsy, in order to further update treatment strategies for refractory epilepsy.

## Data Availability

All data generated or analysed during this study are included in this published article [and its supplementary information files].

## Abbreviations

ASMs: Anti-seizure medications
AEs: Adverse events
BRV: Brivaracetam
CNS: Central nervous system
EEG: Electroencephalogram
FAS: Full analysis set
ITT: Intention-to-treat
ILAE: International League Against Epilepsy
LEV: Levetiracetam
LMICs: Low- and middle-income countries
SV2A: Synaptic vesicle protein 2A
VNS: Vagus nerve stimulation
